# Evaluation of a collaboratively designed digital rehabilitation plan: end users’ evaluating its potential for shared decision-making

**DOI:** 10.1186/s12911-026-03506-0

**Published:** 2026-04-21

**Authors:** Jo Inge Gåsvær, Ilona Heldal, Tobba Sudmann

**Affiliations:** 1 Department for Health and Function, Faculty of Health and Social Sciences, Western Norway University of Applied Sciences, Post Box 7030, Bergen, 5020 Norway; 2 Carasent Norge AS, Oslo, Norway; 3 Department of Computer Science, Electrical Engineering and Mathematical Sciences, Faculty of Engineering and Science, Western Norway University of Applied Sciences, Bergen, Norway

**Keywords:** Digital rehabilitation, Shared decision-making, Patient participation, Patient–provider communication, Implementation, Collaborative design, Rehabilitation plan

## Abstract

**Background:**

Through a partnership and collaborative design process involving technologists and healthcare professionals, a web platform featuring a digital rehabilitation plan was developed. This process tackles recognised challenges in information and communication technology for rehabilitation, such as fragmented data flows and low-quality user interfaces. The digital rehabilitation plan facilitates shared decision-making by allowing patients to customise their plan while providing healthcare professionals with the necessary access to support the patient’s journey. This study assessed the experiences of end-user healthcare professionals and patients regarding the plan’s usability, its potential for shared decision-making, and identified potential areas for improvement.

**Methods:**

This qualitative exploratory study employed semi-structured focus group discussions involving three distinct participant groups: (i) patients, (ii) healthcare professionals, and (iii) a combined group of patients and healthcare professionals. The discussions focused on various stages of the rehabilitation process, including the pre-admission phase, inpatient care, rehabilitation plans, evaluations during inpatient stays, and outpatient follow-ups. The recorded discussions were transcribed and analysed using reflexive thematic analysis to uncover patterns, contradictions, and dilemmas in the participants’ experiences and perspectives.

**Results:**

Patients and healthcare professionals appreciated the digital rehabilitation plan and acknowledged its contribution to shared decision-making. However, patients requested a wider range of digital support and communication tools for inpatient rehabilitation and follow-up, e.g., a “my page” with access to relevant information, chat support, educational tools, and videos. Professionals were reluctant to expand the use of the web platform due to past negative experiences with information and communication technology, and had less motivation to change work processes further. When patients and professionals were brought together, the discussion led to a shift in the professionals’ opinions, revealing a potential for improved collaboration, communication, and shared decision-making.

**Conclusions:**

This study underscores the necessity of involving all stakeholders in the design of a digital tool to ensure that all end-users ' needs and wants are accounted for. Involving patients in both design and implementation can uncover biases, identify barriers, and enhance usability, thereby promoting digital communication and shared decision-making in rehabilitation. Bringing stakeholders together revealed that while healthcare professionals encounter barriers due to established procedures, patients’ familiarity with everyday technology and enthusiasm for digital tools shifted professionals’ perspectives. Lessons learned from this research project were fed back to the developer of the web platform, and taken into account to strengthen the web platform’s potential for communication and shared decision-making in rehabilitation planning.

**Trial registration:**

This study was approved by the Norwegian Agency for Shared Services in Education and Research, Data Protection Services for Research, reference number 116434.

**Supplementary Information:**

The online version contains supplementary material available at 10.1186/s12911-026-03506-0.

## Background

In this paper, we present healthcare professionals’ and patients’ experiences with the development and implementation of a digital rehabilitation plan for an inpatient rehabilitation program. The plan is a component of a configurable web platform that can be customized to meet end-user needs and integrated with electronic health records. Central to the goal of the digital rehabilitation plan was to improve shared decision-making, where healthcare professionals and patients collaborate by sharing the best available evidence to make decisions, while patients are supported to consider options and achieve informed preferences [1].

Upon acceptance into a specialized rehabilitation programme at an institution in Norway, patients begin with a 5-day pre-stay to make plans for a later 3-week full programme. During prestay, they are introduced to the web platform and the digital rehabilitation plan and encouraged to detail their personal goals and means and outline their participation strategies. During the 4-week home period between the pre-stay and main stay, patients are encouraged to further develop their plans. During the main stay, the multidisciplinary team supports the patient by adjusting the plan to ensure that goals and means are relevant to the patient’s functional needs and the rehabilitation institution’s resources.

To maintain patients’ ownership of the digital rehabilitation plan, professionals are not allowed to edit it independently. Professionals have access to digital copies of the plan, which is accessible through the electronic health record, and serves as a point of reference for the professionals. At the end of the main stay, the patient and the multidisciplinary team evaluate goal setting and outcomes together.

This digital rehabilitation plan, and the web platform it is a part of, potentially addresses some of the reported challenges with shared decision-making and communication technology in healthcare and rehabilitation, such as a lack of real-time updated information [2], organizational boundaries (e.g. facilitate collaboration between stakeholders from various organisations) [3], inflexible user interfaces, incompatibility with electronic health records [4–6], and limited opportunities to consider end-user needs [7].

In this study, we acknowledge that patients are rarely involved in the design and implementation of digital communication tools [8, 9] and explore how their needs and preferences can be safeguarded and responded to. Technologists, healthcare providers, and patients may hold differing opinions on the usability and design of a web platform and digital communication for shared decision-making [10]. This study explores how bringing key stakeholders, i.e., technologists, patients, and healthcare personnel, together can create new perspectives on the design and implementation of new digital communication tools for rehabilitation planning. This study is guided by the following question:

How do patients and healthcare professionals evaluate the potential of a digital rehabilitation plan for shared decision-making for the rehabilitation process?

## Methods

A qualitative exploratory design [11, 12] was chosen for this study, grounded in social constructivism. This philosophical stance posits that knowledge and understanding are constructed through active engagement and social interactions [12]. By leveraging this framework, we aimed to explore the complex experiences surrounding the design and implementation of digital tools in rehabilitation. Hence, the choice of a qualitative research design and focus groups comprising both professionals and patients, including mixed groups, was inspired by this stance. Furthermore, social constructivism is reflected in our recognition that the insights shared by patients and professionals were shaped by their unique contexts, relationships, and experiences. This recognition informed our analytical approach, allowing us to interpret the data as a co-construction of knowledge.

All participants provided written informed consent. Norwegian Social Science Data Services (SIKT) approved ethical concerns and privacy protection measures, reference number 116,434.

### Participants and recruitment

Health care professional participants were approached and informed by leaders at an inpatient rehabilitation institution. They were given oral and written information about the study. The professionals who accepted to take part in the study were asked to inform and invite patients to take part in the study. In total, the participants included ten persons from Norway familiar with the digital rehabilitation plan; six patients (Table 1) and four healthcare professionals (Table 2).

**Table 1:** Patient characteristics

Gender	Age	Education	Profession	Type of rehabilitation	Work status
Female	35	High school	Healthcare worker	Vocational	Sick leave 100%
Female	30	Primary school	Shop worker	Vocational	Work assessment allowance
Male	25	High school	Electrician	Vocational	Sick leave 100%
Female	60	Primary school	Kindergarten assistant	Vocational	Work assessment allowance
Female	45	High school	Health secretary	Cancer	Disability benefit 80%
Female	50	University	Environmental healthcare professional	Cancer	Sick leave 30%

**Table 2:** Healthcare professional characteristics

Gender	Age	Profession	Expertise
Female	40	Nurse	Cancer nursing
Female	25	Physiotherapist	Master Health Sciences
Female	55	Nurse	Cognitive Behavioral (CBT)- and Acceptance and Commitment (ACT) Therapy
Female	50	Social worker	Personal economy, CBT, ACT

### Data production

Semi-structured focus mini-group discussions [13] were conducted with pairs of (i) patients, (ii) healthcare professionals, and (iii) a combination of both groups. In total, six patients and four healthcare professionals were interviewed. Two of the professionals served as patient coordinators for four of the patients. In total, eight focus groups were conducted, consisting of the following configurations: three groups of patient pairs, two groups of healthcare professional pairs, and three groups combining patients and their affiliated professionals.

Semi-structured discussions were chosen because of the method’s flexibility, where predefined themes are set; however, further exploration can be performed based on participants’ answers. Open-ended questions and prompts enabled the interviewees to disclose their knowledge, experiences, and perspectives. This is useful in an exploratory study where understanding nuanced perspectives is important.

The venue for focus groups was provided by the rehabilitation institution. The group discussions followed a topic guide (supplementary 1). The digital rehabilitation plan is designed to facilitate shared decision-making in planning, implementation, and evaluation of the rehabilitation trajectory. The present research project is deliberately designed to enhance patients’ voices and to ensure that their experiences, opinions, and suggestions are taken into consideration in further development and implementation of the web platform. Henceforth, focus groups with patients were carried out before focus groups with healthcare personnel. Lessons learned in focus groups with patients were used as prompts in focus groups with healthcare personnel, and lessons learned in both groups were used as prompts in the joint focus groups with patients and professionals brought together. Each group discussion lasted between 60 and 90 min and was audio recorded with a Dictaphone.

### Data analysis

The focus group discussions were transcribed verbatim in Norwegian by JIG and anonymized continuously. The transcripts were categorized via NVivo 14 software (QSR International Pty Ltd., Victoria, Australia). The initial codes were grouped into broader themes and then further reviewed and refined by rechecking them against the original transcripts to ensure they reflected the dataset (see Table 3 for examples on the relations between key codes/sub themes, themes and findings). Special consideration was given to balancing the use of codebooks and ensuring coding reliability while embracing the subjective nature of reflexive thematic analysis [14]. Excerpts from the discussions used in this paper were translated into English and edited for readability by the authors.

**Table 3:** Examples of the analysis process

Theme	Subtheme/Key codes	Findings
Positive views on digital rehabilitation	Overall positive perception by patients	Patients and healthcare professionals held positive views of the digital rehabilitation plan
	Recognition of the plan as a dynamic document	Patients appreciated the ability to revise goals and plans flexibly
	Enhanced patient engagement through usability	Healthcare professionals found the plan supportive of treatment processes
Challenges with the digital rehabilitation plan.	Stress related to the first-day submission	Patients: there is no comprehensive digital approach to support the rehabilitation process
	Difficulty in initiating the rehabilitation plan	Patients felt unprepared to take an active role in their rehabilitation
	Concerns over lack of follow-up from professionals	Patients sought more extensive support after the plans initial creation.

## Results

We employed a multifaceted analytic strategy in this study to gain a nuanced understanding of participants’ perspectives on digital rehabilitation plans, highlighting multifaceted experiences. We first analysed the perspectives of patients, and then the professionals’ perspectives, to identify unique experiences, desires, and internal variations. This approach allowed us to uncover specific errors, gaps, suggestions for improvement, and areas of contention within each group’s narratives. We subsequently examined the commonalities and differences between the patients and the providers to determine where their interests aligned, where disagreements arose, and how they might ultimately converge on shared insights. In the analysis of the third focus group, where patients and professionals were brought together, we looked for deliberation and discussions that made participants change their minds, align their arguments, or were at odds with each other. This allowed for a multifaceted understanding of the participants’ views on the digital rehabilitation plan (3.1), stakeholders’ experiences with digital solutions that support the rehabilitation process in general (3.2 and 3.3), and the discovery of further technological solutions needed to work toward digital support for shared decision-making (3.4).

###  Patients and healthcare professionals held positive views of the digital rehabilitation plan

Both healthcare professionals and patients conveyed a generally positive perception of the digital rehabilitation plan. The patients unanimously approved the digital rehabilitation plan for setting goals and means. They underscored their own tech-savviness, raised concerns about demands for e-health literacy, and recommended that paper-based rehabilitation plans should be available when needed.

*You get a bit of pressure when you must write on paper..*,* you kind of feel like you just must get something dotted down. But when you do this on the web platform*,* you can go back later and finish it or change it. It makes it a lot easier - patient*

Healthcare professionals viewed the digital rehabilitation plan as a support tool for the treatment process. They recognised that the plan functioned as a “living document” that could be dynamically updated, enhancing both patient engagement and document usability. The digital plan supported the physical meetings, and the professionals became better at using the digital solutions through increased use. Healthcare professionals identified several barriers related to the user interface, including the absence of real-time notifications for updates, the availability of templates exclusively in Norwegian, and challenges associated with navigating various user interfaces.

*I guess it depends a bit on age*,* too. For young people*,* it’s not a problem. However*,* for those who are 60-plus*,* maybe. In addition*,* some have problems with their cell phone*,* so they cannot access the web platform. We believe that this might be related to outdated software or older devices*,* but we do not know. I do not always understand that - professional*

### Patients’ evaluation of the digital rehabilitation plan: challenges, suggestions, and overall satisfaction

Patients are required to submit the digital rehabilitation plan on their first day of pre-stay at the rehabilitation institution. For the patients, this was at odds with previous experiences from healthcare encounters and inpatient stays where treatment decisions were largely made by professionals. Being active and taking initiative, hence, was unfamiliar. As a result, they struggled to initiate the rehabilitation plan.

*I felt a lot of stress upon arriving at the institution and in the first few days. I didn´t know where I was going*,* what to expect*,* or what the treatment consisted of. In addition*,* since I was accustomed to just being told what to do*,* the concept of overseeing my rehabilitation plan was difficult… I also struggle with memory and cognition*,* so even though I was given information about how to work with the rehabilitation plan*,* I could neither remember nor do it myself - patient*

The customization of the digital rehabilitation plan positively influenced patients’ experiences. However, patients were surprised that healthcare professionals did not provide follow-up on the plan as extensively as anticipated following its mandatory creation. For them, this lowered its potential for shared decision-making.

Furthermore, the patients suggested several new ways of using digital tools to improve their rehabilitation experience. As tech-savvy users, they sought access to digital information before the inpatient period, including videos and details about the institution and directions for arrival. They proposed a “my page” feature that would provide essential information, training programs, survey responses with explanations of the results, timetables, appointments, and tasks. Additionally, they expressed concern about the period following their stay and requested digital follow-up, self-management support, and opportunities for contact with the rehabilitation institution as needed. Shared decision-making necessitates two-way communication.

*It is a lot that is connected to the rehabilitation plan. Lectures*,* appointments*,* handouts*,* and everything… With my fatigue and memory loss*,* everything becomes more complicated*,* and it is hard… - patient*

Patients were highly satisfied with the inpatient program, valuing both the collaboration with professionals and the peer support provided by fellow patients. They expressed strong scepticism regarding the idea of replacing inpatient rehabilitation with digital follow-up alone. Instead, they advocated for digital solutions that could support and enhance, rather than replace, the existing rehabilitation process.

### The professionals’ evaluation of the digital rehabilitation plan: “We do as we usually do, even though we like it”

Healthcare professionals expressed appreciation for the reduced administrative burden that the digital rehabilitation plan represented. They highlighted benefits such as less paperwork; printing, punching patient input, scanning documents to the EHR, and time saved for looking for paper versions. However, despite recognizing these advantages, the professionals also voiced scepticism regarding the full integration of technology into their treatment approach, justified with reference to work habits and professional code of conduct in health-related encounters. Professionals stressed that patient‒provider interaction must be analogous and warned that digital solutions could hamper this. The professionals did not discuss how other digital solutions could support communication, shared decision-making, and the patients’ rehabilitation experience.

*I feel that bringing a PC into patient meetings can be a bit disrespectful. Therefore*,* when I meet with patients*,* I prefer to have important points written down in advance. I’m also concerned that discussing things digitally might lead to missing crucial information during conversations - professional*

###  “When healthcare professionals and patients understand each other, they can explore possible solutions”

The dialogue between patients and healthcare professionals changed professionals’ perspectives after patients shared their experiences with unsatisfactory digital support throughout the rehabilitation process. First, the professionals acknowledged challenges in effectively collaborating with patients on the digital rehabilitation plan.

*We have the rehabilitation plan up on the wall in the team meetings*,* but maybe we are not transferring this clearly enough to the patient when we talk with them - professional*

Second, the healthcare professionals acknowledged that they did not routinely check whether patients’ progress was according to plan. Thirdly, the healthcare professionals noted a lack of coordination between the development of the rehabilitation plan and clinical treatment objectives. This disconnect led stakeholders to focus more on the treatment process, resulting in difficulties integrating it into the rehabilitation plan.

In this joint group, professionals reflected more on how technology could enhance their work compared to the perspectives shared in the professionals-only group. They became responsive to the patients’ wants and their suggestions for ICT to facilitate communication between patients and providers, as well as for planning, evaluation, and feedback from assessments:

*We find it difficult to absorb all the information in the first days of the stay; therefore*,* it is hard to make a rehabilitation plan” - patient*.

*…but we bring it up at the welcome meeting - professional*

*Yes*,* but then we have already arrived - patient*

*Yes*,* you just get thrown into everything*,* and then it is straight into individual meetings with the professionals*,* and from then on*,* it is a rush to get through everything at the start of the stay. So yes*,* I can see that it can be a lot for you - professional*

Further negotiations and possible solutions emerged through these discussions, which focused on patient engagement and collaboration:

*When talking about the new possibilities*:

*All the data we collect*,* it must be of importance to you*,* and we must explain more to you… To see development on scores can be valuable for the patient – have I become more robust? - professional*

*Yes*,* we want to be able to compare - patient*

*Yes*,* that is exciting*,* both for us*,* you*,* and for research… It is very good that this comes up*,* it is new for us – we are in a transition process - professional*

In contrast to their experiences at the rehabilitation centre, where patients reported strong feelings of trust and teamwork with the professionals, they expressed that these feelings were less pronounced in their interactions with their general practitioners (GPs) and the broader healthcare system. The healthcare professionals acknowledged this sentiment and discussed the potential of utilizing digital tools to strengthen collaboration with patients.

These results illuminate the intricate dynamics of the rehabilitation process and digital tools to support it. While both patients and professionals expressed satisfaction with the digital rehabilitation plan itself, the patients expressed difficulties in initiating and engaging with the plan as well as suggesting possible solutions using digital tools. In contrast, professionals hesitated to fully embrace the plan and digital tools in their practice, offering a preference for face-to-face encounters for communication and shared decision-making. However, when presented with the patients’ arguments, the professionals became more receptive to further developing the digital rehabilitation plan and exploring additional digital tools to enhance the possibilities for using digital tools to promote shared decision-making as a supplement to face-to-face encounters.

## Discussion

Anchored in social constructivism and employing a multifaceted analytic strategy to explore the complex experiences surrounding the design and implementation of technological solutions in rehabilitation, this study revealed a high level of appreciation for a digital rehabilitation plan among patients and healthcare professionals. However, patients experienced challenges related to the implementation of the digital rehabilitation plan and unsatisfactory follow-up from the providers’ side. Healthcare professionals responded to this need during discussions with patients, demonstrating both troubleshooting skills and enthusiasm. They engaged with patients, reflecting on the use of the digital rehabilitation plan for shared decision-making and the practical possibilities with technology.

### At first, eyesight: the digital rehabilitation plan is a success!

The implementation of a digital rehabilitation plan was well received by both patients and healthcare professionals, reflecting the rehabilitation institution’s commitment to fostering shared decision-making, which is defined as an approach where professionals and patients share the best available evidence to make informed and shared decisions, and where patients are supported to consider options to achieve informed preferences [1]. This positive reception is consistent with the literature, which underscores the importance of involving key stakeholders in the design and deployment of technological solutions to ensure that they effectively meet the needs of end-users [7]. Even though data access and, hence, participation for other relevant stakeholders (e.g., GPs, next of kin, employers) was not possible (e.g. due to legal restrictions related to data access, organizational boundaries) [15], at least access to pertinent information was possible for patients and healthcare professionals inside this part of the health service [2], enabling effective use of the digital rehabilitation plan.

### Shared decision-making through technology is dependent on both technology and end-users

In our study, patients highlighted several barriers that obstructed the effectiveness of the digital rehabilitation plan. These challenges arose from a disconnect between individual patient experiences and the standardized healthcare framework that guided the design of the system [6, 16, 17]. Due to the patients’ prior experiences of having healthcare professionals make decisions regarding their treatment in other areas of the healthcare system, their autonomy in initiating their own rehabilitation plans was constrained.

Furthermore, cognitive and memory challenges reported by the patients in this study impeded the ability to develop personalised strategies, thereby undermining the principles of shared decision-making. Executive functions, including planning, memory, problem-solving, and advanced reasoning, can be affected to varying degrees by factors such as chemotherapy, trauma, and complex long-term health issues, which are often the reasons patients require rehabilitation. These challenges can pose obstacles for patients and are recognized as critical barriers to achieving effective co-creation between patients and healthcare professionals in the context of long-term health issues and rehabilitation [4].

The current healthcare landscape has led to limited collaboration between primary and specialist care [3, 6]. This was evident, as healthcare professionals often overlooked the challenges patients faced, highlighting a discrepancy in perspectives. While patients seek assistance regardless of organizational constraints, professionals focus on care within their established boundaries [3, 7]. This divergence complicates the creation of digital support tools that address patient needs, as solutions favored by professionals may not align with patient desires. This can ultimately undermine collaboration and effective shared decision-making. Furthermore, the reliance on paper-based information sharing and fragmented digital communication exacerbates this divide.

This highlights the complexities in technology design for rehabilitation settings, where heterogeneous stakeholders can possess diverse values, ambitions, and perspectives [5].

###  Joint conversations uncover insights into the role of digital solutions in shared decision-making

An interesting finding in this study was the shift in attitudes and focus when healthcare professionals and patients were brought together in a focus group discussion. As in the patient-only focus group, the patients shared their reflections on the challenges they faced in initiating the digital rehabilitation plan and dealing with other cumbersome solutions. They proposed leveraging technology to enhance patient engagement and deliver integrated, proactive healthcare with digital support [18]. The shift in the professionals’ perspectives during joint discussions fostered a sense of togetherness, allowing professionals to better understand patients’ experiences and needs. The discussions shifted the providers’ opinions to acknowledge the added value of the digital rehabilitation plan in face-to-face encounters and in the overall outcome of the rehabilitation process. This collaborative approach provided patients with opportunities to voice their opinions and experiences and encouraged professionals to consider new perspectives on care delivery, going beyond their existing context and boundaries, and partnering to promote a patient-centred focus on technology supporting the rehabilitation process. This underscores the vital importance of involving all end users, not only professionals, in the design and implementation of a digital rehabilitation plan. The joint meetings between patients and healthcare professionals were instrumental in uncovering insights that might have remained obscured in separate discussions. Without these sessions, the study would likely have highlighted only the existing discord between patients and professionals regarding their perceptions of the challenges and opportunities presented by ICTs in the rehabilitation process. However, these dialogs fostered a deeper understanding among professionals, empowering patients to influence the future design of ICT solutions that support the rehabilitation process [8, 9].

### Collaboration in technology for rehabilitation must involve end-users

Given our findings, we advocate greater end-user involvement in the design process. Such engagement could lead to improvements in digital support services and transform the rehabilitation framework [16, 17]. Firsthand experiences and insights from patients are invaluable for creating effective, user-friendly health technologies. Furthermore, patients, drawing on their familiarity with technology across various aspects of their lives, are ideally positioned to offer innovative solutions to the identified challenges and enhance the services provided. Therefore, it is essential to recognize patients as key stakeholders and actively invite them to participate in the collaborative design and implementation of healthcare technology. Shared decision-making is not placation; it is real-life collaboration and communication.

While our findings highlight the potential added value of involving all stakeholders in the design of the rehabilitation plan, several barriers persist. The context of technology and healthcare is influenced by factors such as mergers, acquisitions, shifting political priorities, and budget constraints. At the local level, tender regulations can complicate collaborative efforts, often limiting the meaningful participation of patients. In many cases, patients are either excluded from the stakeholder process altogether or participation is placation or manipulation [19], where user representatives’ participation is illusory [20]. Finally, local adaptations and customisation of technology require substantial resources from technology designers and may not yield the anticipated economic benefits [21]. The siloed structure of the Norwegian healthcare system, characterised by incompatible administrative and health-related systems, creates barriers to data access and limits stakeholders’ digital engagement in the design, implementation, and everyday use of technology.

## Conclusion

In conclusion, this study highlights patients’ and end-user professionals´ appreciation for a digital rehabilitation plan to promote shared decision-making among end-users and uncover barriers in design and day-to-day use. The societal and economic context of a rehabilitation institution creates barriers to furthering a collaborative process of design and local adaptations. The focus group discussions between patients and healthcare professionals provided a window of opportunity for learning and sharing knowledge and experiences, prompting professionals to shift their perspectives toward the importance of integrating patients’ knowledge and ideas into the design and use of technology. A collaborative design process may facilitate new usage patterns and ideas for further development. When unforeseen capabilities or applications of the technology are identified, the implicit design script [16, 17] can be updated. However, siloed healthcare organisations and regulatory constraints can still hinder the implementation and day-to-day use of any technology. Despite the identified barriers to acknowledging and including patients as stakeholders in design and implementation processes, there is no other alternative if patient-centredness is to be obtained.

### Limitations

Semistructured mini-focus group discussions were chosen to foster interaction among participants and to explore diverse perspectives and generate ideas within and between stakeholder groups. However, this method has several challenges. Dominant personalities can skew discussions, and in groups with up to 12 participants, conformity pressure can inhibit the expression of differing perspectives [22]. To mitigate the impact of such processes, the focus groups size were small, the moderator ensured that all participants were engaged in the discussion, and the moderator shared lessons learned from previous groups, from clinical practice, and from the literature to prompt discussion when needed. Focus groups facilitate deliberation to a greater extent than individual interviews do, which is demonstrated by the reported change in positions through the discussions.

The first author of this study declares a vested interest, as he is affiliated with the design company. However, the active participation of coauthors/senior researchers (social scientists and computer scientists) contributed to a critical appraisal of the study design, material, analysis, and knowledge contribution.

## Supplementary Information

Below is the link to the electronic supplementary material.

Supplementary Material 1

## Data Availability

Data supporting this study cannot be made publicly available owing to the risk of breaking anonymity among study participants.

## Declarations

### Concent for publication

Verbal and written informed consent were obtained from the participants for their anonymized information to be published before they were interviewed.

## Abbreviations

ACT: Acceptance and Commitment Therapy
CBT: Cognitive Behavioral Therapy
GP: General Practitioner
ICT: Information and Communication Therapy
SIKT: The Norwegian Social Science Data Services
