# Supplementary material for: Evaluation of a collaboratively designed digital rehabilitation plan: end users’ evaluating its potential for shared decision-making
Source: BMC Med Inform Decis Mak. 2026 Apr 21;26:202. doi: 10.1186/s12911-026-03506-0 (PMC13231764; doi:10.1186/s12911-026-03506-0)
Supplement: Supplementary file 1 — Supplementary Material 1 [file 12911_2026_3506_MOESM1_ESM.docx]

# Interview guide

## Patients and therapists' experience with the use of digital solutions to support collaboration on goals and measures, provide decision support, and support to evaluate the effect of the rehabilitation pathway

Topic:

Patients and therapists’ experience with the use of a web platform as digital support for collaboration on goals and measures, decision support, and evaluation of rehabilitation pathways.

Purpose:

Gain more knowledge about experiences with the use of the web platform for the collaboration process.

Conducting the focus group interview:

The interviews will be conducted at a time and place that suits the participants. Review of information letter and consent form, interview, and conclusion takes 1.5-2 hours.

A digital audio recorder will be used during the interview (not in the initial conversation and rounding up) if the participant agrees to this.

Audio will be stored on a research server at Western Norway University of Applied Sciences and deleted from the audio recorder.

1. Introduction to the study

The interviewer begins by introducing himself and providing an overview of the purpose of the interview. He emphasizes the significance of the participant's insights and explains how the information gathered will be utilized. Additionally, he underscores the commitment to maintaining confidentiality and anonymity, assuring participants that their responses will be handled with the utmost care and will remain private

Participants then introduce themself.

1. Short biography

Name

Age rounded to the nearest 5/10 years

Gender: she, he, hen, will not disclose

Education, work, and position:

Patient: admitted for rehabilitation (health service):

Earlier experiences from the health sector and other areas that the participants find relevant for their role today

1. Information (the below shall not be read aloud, it is meant to remember key elements). Some themes around the topic of the conversation (background, purpose)

- A web platform has been developed in close collaboration between IT developers and customers. It is technologically ready, and one has implemented some functionality for the customers.
  - In theory, this is a smart way to do it, since the customers know health and have provided the developers with good advices along the way, and tested the solutions as they have been designed. And now we are going to get the experiences from those who actually use the designed solutions about your experiences so far.
- The actual use and experience are important for several reasons. We get to evaluate the technology - which is my research. But we also hope to get tips for further development as well.

1. Introductory questions

Based on your background/role, can you share your experiences from using this web platform for collaboration? When was the first time you heard about it? When did you use it for the first time? Have you used it?

1. Key questions
2. Focus on the collaboration, the job with goals/measures, decision support, and possibly evaluation of treatment.

Patient: How has it been working with goals and measures, making decisions about the rehabilitation process, and possibly evaluating whether they achieved goals? How is this different from doing it paper-based, if you had to do that?

Healthcare personnel: You have done this using pen and paper earlier. What is the biggest difference when collaborating with the patient on the rehabilitation plan digitally? (e.g. focus on digital versions of the plan, working back and forth in the plan). What are your experiences with the web platform so far?

Joint patient/healthcare personnel: What has it meant for the collaboration/the common understanding to work digitally as you have done with this solution?

1. Have you got specific feedback on functionality that is good, and functionality you feel is lacking? Both user-friendliness/user experience and any new features are relevant.

Patient: You are at the center of your own treatment; it is both a right you have and the most effective for the result of the treatment. What functionality has been helpful? Are there functions you feel that we should add to the solutions to make them better? Have you thought about something that I should report back to those who make this?

Healthcare personnel: What is good about collaborating digitally in this way? Is there something that feels simpler and gives better quality assurance (e.g., doing it digitally, single sign-on, the versions of the plan in the electronic health record)? Do you miss something, or do you have tips for something we can improve? Have you thought about things that I should report back to those who make this?

Joint patient/healthcare personnel: What do you think is good about working together digitally? Have you got suggestions for improvement? Are there things I should report back to those who make this?

1. Specific matters that may be relevant to discuss if it has not come up before:

Training

Security/Privacy

Technical conditions

Have you heard others who have talked about this?

1. Summary

Summarize the group conversation (can also be done along the way)

What I have noticed in our conversation that was particularly interesting to me was ....

Have I understood you correctly when I say...?

Is there anything you have thought about before the conversation today that you want to tell me?

Or maybe you have thought about something that I haven't asked you about?

I will continue with this PhD work for another year, are there any people you think I should talk to or visit?

Do you have suggestions for what I should ask the next participants about?
